# Supplementary material for: Utilization of the Rat Tibial Nerve Transection Model to Evaluate Cellular and Molecular Mechanisms Underpinning Denervation-Mediated Muscle Injury
Source: Int J Mol Sci. 2024 Feb 3;25(3):1847. doi: 10.3390/ijms25031847 (PMC10855399; doi:10.3390/ijms25031847)
Supplement: Supplementary file 1 [file ijms-25-01847-s001.zip › ijms-2815381-supplementary.pdf]

Supplemental Materials and Methods

Utilization of the Rat Tibial Nerve Transection Model to evaluate cellular and molecular mechanisms underpinning denervation-mediated muscle injury.

Christina Doherty[1], Monika Lodyga[1], Judy Correa[1], Caterina Di Ciano-Oliveira[1], Pamela Plant[1], James Bain[2], Jane Batt[1,3]

1) Keenan Centre for Biomedical Research of Saint Michael's Hospital, Unity Health Toronto, Ontario, Canada
2) McMaster University Faculty of Health Science, Division of Plastic Surgery, Hamilton, Ontario, Canada
3) Department of Medicine, Temerty Faculty of Medicine, University of Toronto, Toronto, Ontario, Canada

FAPs Cell Culture

Media

1) FAPs Basal Growth Media

- 90% Base Media:
  - 79% DMEM DMEM [Gibco, 11995-065]
  - 20% FBS FBS [Sigma, F1051]
  - 1% Penicillin-Streptomycin Millipore Sigma, P4333]
- 10% Heat-inactivated Horse Serum [Gibco, 26050-088]
- 2.5 ng/mL basic Fibroblast Growth Factor (bFGF) [Gibco, PHG0266]

2) FAPs Adipogenic Differentiation Media:

- 78% DMEM
- 20% FBS
- 1% Penicillin/Streptomycin
- 1.25 µM Dexamethasone (stock concentration 5 mg/mL) stock solution Millipore Sigma, D4902]
- 0.5 mM IBMX (stock concentration 10 mg/mL) Millipore Sigma, I5879]
- 5 µM Troglitazone stock solution (stock concentration 1 mg/mL) [Millipore Sigma, T2573]
- 1 µg/mL Humulin R (stock concentration 1 mM) [Lilly, HI0210]

RT qPCR Primers

Rat Primers

Gene	Forward Primer	Reverse Primer
Perilipin-1	GTGGCTCTCAGCTGCATGT	TTCTGGAAGCACTCACAGGTCC
GDNF	GCGGTTCCTGTGAAGCGGCCGA	TAGATACATCCACACCGTTTAGCGG
PCNA	CGTCGCAACTCCGCCACCAT	TCACGCCGCCCGAACTGATG
SMA	CCAGCCAGTCGCCATCAGGA	GCCCGGAGCCATTGTCACAC
Col1a1	AAAACGGGAGGGCGAGTGCT	CTCCCTTGGGTCCCTCGACT
Ki67	CTGCAGAGAAGGTTGGGATAAA	CTGACTTTGCCCAGAGATGAA
HPRT	GCCGACCGGTTCTGTCAT	TCATAACCTGGTTCATCATCACTAATC
HMBS	GGCTCAGATAGCATGCAAGAGA	TGGACCATCTTCTTGCTGAACA
GAPDH	GACCACAGTCCATGCCATCACTGC	GCTGTTGAAGTCGCAGGAGACAAC
